# The FAM13A Long Isoform Regulates Cilia Movement and Coordination in Airway Mucociliary Transport

**DOI:** 10.1165/rcmb.2024-0063OC

**Published:** 2024-05-01

**Authors:** Ashleigh Howes, Clare Rogerson, Nikolai Belyaev, Tina Karagyozova, Radu Rapiteanu, Ricardo Fradique, Nicola Pellicciotta, David Mayhew, Catherine Hurd, Stefania Crotta, Tanya Singh, Kevin Dingwell, Anniek Myatt, Navot Arad, Hikmatyar Hasan, Hielke Bijlsma, Aliza Panjwani, Vinaya Vijayan, George Young, Angela Bridges, Sebastien Petit-Frere, Joanna Betts, Chris Larminie, James C. Smith, Edith M. Hessel, David Michalovich, Louise Walport, Pietro Cicuta, Andrew J. Powell, Soren Beinke, Andreas Wack

**Affiliations:** ^1^ Immunoregulation Laboratory,; ^2^ Protein-Protein Interaction Laboratory,; ^3^ Developmental Biology Laboratory, and; ^4^ Bioinformatics and Biostatistics, The Francis Crick Institute, London, United Kingdom;; ^5^ Immunology Research Unit,; ^6^ Crick-GSK Biomedical LinkLabs,; ^7^ Functional Genomics,; ^8^ Protein, Cellular and Structural Sciences,; ^9^ Computational Biology, and; ^10^ Refractory Respiratory Inflammation Discovery Performance Unit, GSK R&D, Stevenage, United Kingdom;; ^11^ Cavendish Laboratory, University of Cambridge, Cambridge, United Kingdom;; ^12^ Capgemini Engineering, Capgemini UK, Stevenage, United Kingdom; and; ^13^ Development Digital and Tech, GSK, Collegeville, Pennsylvania

**Keywords:** multiciliated cells, COPD, cilia movement, mucociliary clearance, airways

## Abstract

Single nucelotide polymorphisms (SNPs) at the *FAM13A* locus are among the most commonly reported risk alleles associated with chronic obstructive pulmonary disease (COPD) and other respiratory diseases; however, the physiological role of FAM13A is unclear. In humans, two major protein isoforms are expressed at the *FAM13A* locus: “long” and “short,” but their functions remain unknown, partly because of a lack of isoform conservation in mice. We performed in-depth characterization of organotypic primary human airway epithelial cell subsets and show that multiciliated cells predominantly express the FAM13A long isoform containing a putative N-terminal Rho GTPase-activating protein (RhoGAP) domain. Using purified proteins, we directly demonstrate the RhoGAP activity of this domain. In *Xenopus laevis*, which conserve the long-isoform, Fam13a deficiency impaired cilia-dependent embryo motility. In human primary epithelial cells, long-isoform deficiency did not affect multiciliogenesis but reduced cilia coordination in mucociliary transport assays. This is the first demonstration that FAM13A isoforms are differentially expressed within the airway epithelium, with implications for the assessment and interpretation of SNP effects on *FAM13A* expression levels. We also show that the long FAM13A isoform coordinates cilia-driven movement, suggesting that *FAM13A* risk alleles may affect susceptibility to respiratory diseases through deficiencies in mucociliary clearance.

**Clinical Relevance**

Here, we show that the chronic obstructive pulmonary disease (COPD) genome wide association study hit *FAM13A* is a gene that exists in two isoforms, of which only the longer has RhoGAP activity and is specifically expressed in multiciliated cells of the airways. We demonstrate in human primary airway cultures *in vitro* and in a *Xenopus laevis* *in vivo* model that the long isoform of FAM13A is a key regulator of cilia coordination, required for mucociliary transport. Therefore, we have identified a novel physiological role of this gene, indicating that *FAM13A*, most likely acting through its RhoGAP function, coordinates cilia function in multiciliated cells, with strong implications for COPD aetiology.

Chronic obstructive pulmonary disease (COPD) is a progressive disease characterized by airflow limitation in the lungs. Key environmental risk factors for COPD are tobacco smoking and, in developing countries, exposure to indoor pollutants generated by burning biomass fuels. Mechanistically, the factors driving COPD are complex, involving dysfunctional airway epithelial repair and defense processes, as well as chronic inflammation (1, 2). Current COPD therapies relieve symptoms but ultimately do not stop disease progression, as they fail to target underlying pathogenic mechanisms. As COPD is estimated to be the third leading cause of death worldwide, according to the World Health Organization (https://www.who.int/respiratory/copd/en/), there is a clear need to increase our understanding of COPD pathogenesis to facilitate the development of more effective treatments.

Genome-wide association studies have identified several single nucleotide polymorphisms (SNPs) that affect COPD susceptibility. Among the most frequently reported SNPs are those within the *FAM13A* (family with sequence similarity 13, member A) gene (3). In humans, distinct promoters give rise to the expression of several *FAM13A* transcripts consisting of one full-length and at least four highly similar truncated isoforms (4). The full-length or “long” isoform of FAM13A contains a putative N-terminal Rho GTPase-activating protein (RhoGAP) domain and two C-terminal coiled-coil domains. In contrast, the “short” FAM13A isoforms retain only the coiled-coil domains, raising the possibility that FAM13A isoforms have both differential expression patterns and potentially distinct functions.

COPD-associated *FAM13A* SNPs lie within early intronic regions of the gene, and their noncoding nature implies that they have regulatory consequences. Several reports have demonstrated a tendency for increased overall *FAM13A* mRNA expression in lung tissue of risk allele carriers (5–7). In agreement, massively parallel reporter assays suggest a role for COPD risk alleles in driving *FAM13A* expression (8). However, it is important to note that these studies have been conducted on whole-lung tissue and have only looked at *FAM13A* expression at the gene level. As such, the effect of these SNPs on expression of individual *FAM13A* isoforms remains unknown.

The airway epithelium is primarily composed of self-renewing basal cells, mucus-producing secretory cells, and multiciliated cells. These cells coordinate mucociliary clearance, which traps and removes particles, including pathogens, from the lung. Airway epithelial cells are also central in sensing and initiating responses to environmental factors such as pollutants, infection, or injury (9). Because of their known roles in contributing to COPD pathogenesis, the majority of FAM13A functional studies have focused on these cell types.

Using human lung cancer cell lines and *Xenopus* embryo overexpression studies, Jin and colleagues showed that FAM13A could activate the WNT signaling pathway (10). However, conflicting studies using human bronchial epithelial cell lines and murine *in vivo* challenge models reported that FAM13A inhibits WNT signaling through the destabilization of β-catenin (11, 12). In a human airway cell line, FAM13A deficiency also increased β-catenin expression but was linked to increased transforming growth factor (TGF)-β1–mediated epithelial remodeling rather than WNT signaling (13). Independently, FAM13A upregulation was also linked to increased TGF-β1–mediated epithelial-to-mesenchymal transition (EMT) of human small airway cells (14). In contrast, in mice, *Fam13a* deficiency increased EMT in the lung (15). Distinct from any effects on WNT signaling, in human lung epithelial cell lines and primary murine lung epithelial cells, FAM13A has also been linked to TGF-β2 secretion (16) and reported to promote fatty acid oxidation. This was proposed to mediate airway epithelial damage by means of increased production of reactive oxygen species and cell death (17). Most recently, FAM13A protein levels were shown to be regulated by AKT1-dependent phosphorylation in human cells and mice, with phosphorylation-dependent degradation leading to increased epithelial cell proliferation in mice (18).

Although these studies begin to dissect the mechanisms by which FAM13A could contribute to COPD pathogenesis, many of them are limited by a reliance on murine models which, because of a 5′ rearrangement in the *Fam13a* gene, express only the short isoform of *Fam13a* (www.ensembl.org/Mus_musculus/Transcript/Summary?db=core;g=ENSMUSG00000037709;r=6:58909075-59001534;t=ENSMUST00000089860) (19), limiting the relevance of these results for human FAM13A function. This rearrangement is not widespread, however, as frogs and zebrafish (among other species) have conserved the long isoform of FAM13A (Figure 1A). This fundamental difference, which has yet to be addressed in the literature, indicates that mice are, in fact, a model for the function of the short isoform of FAM13A only. Furthermore, where human cell lines have been used for knockdown studies, an analysis of FAM13A isoform-specific expression has not been performed. Thus, although conservation of the short isoform suggests a meaningful biological function, in the human system—where both isoforms are present–it is not clear whether expression of the FAM13A long isoform impacts on WNT signaling, fatty acid oxidation, or an undescribed alternative RhoGAP-mediated activity.

**Figure 1.**
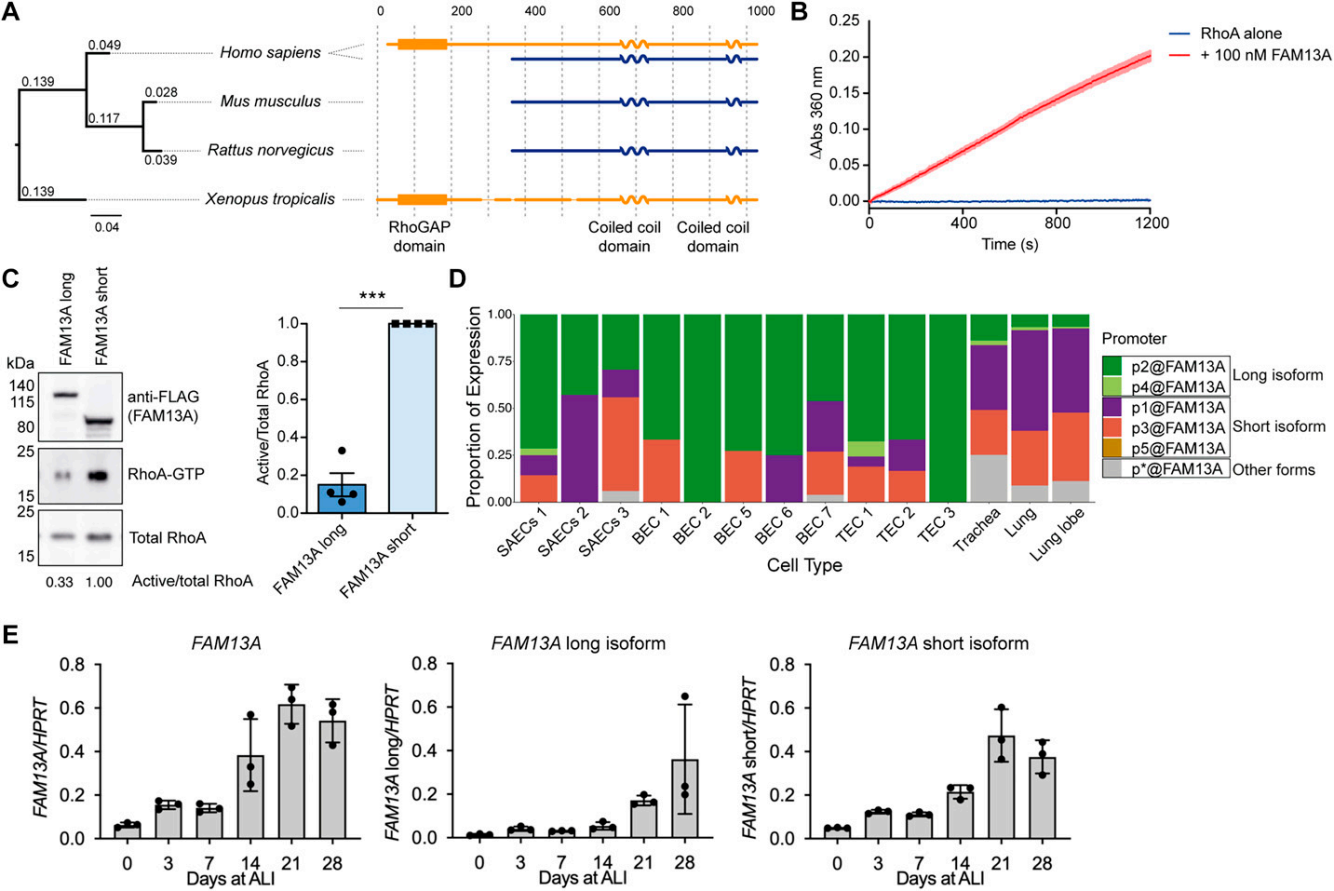
FAM13A isoforms are differentially expressed across species and between human airway epithelial cell types. (*A*) Representation of the conservation of the two FAM13A isoforms across human, mouse, rat, and frog species. Distances are based on the conservation of the region shared between the long and short isoforms. Length of solid black lines demonstrates number of substitutions per site, bar of length 0.04 shown for scale. (*B*) GTP hydrolysis of purified RhoA alone or incubated with 100 nM purified FAM13A N-terminal domain. Δ Abs represents change in absorbance. (*C*) Pulldown of active RhoA from HEK 293T cell lysates with overexpressed FLAG-tagged FAM13A long isoform or FAM13A short isoform and quantification of the ratio of active RhoA-GTP to total RhoA, normalized to the levels when FAM13A short isoform is expressed (*n* = 4, paired *t* test, *P* = 0.0008). (*D*) Abundance of *FAM13A* isoform expression in lung tissue and airway epithelial cell types in the Functional Annotation of the Mammalian Genome 5 data set. (*E*) mRNA expression levels of *FAM13A* isoforms during primary human bronchial epithelial cell (hBEC) differentiation at indicated days of ALI culture. Values are normalized to *HPRT1*. Bars represent triplicate cultures and mean ± SD. BEC = bronchial epithelial cells; RhoGAP = Rho GTPase-activating protein; SAEC = small airway epithelial cells; TEC = tracheal epithelial cells.

Few studies have specifically addressed the isoforms of FAM13A. In a human lung cancer cell line, *FAM13A* long and short transcripts were detected, and in lung tissue samples, expression of the short isoform correlated with levels of hypoxia-inducible factor 1α (20), suggesting a link between FAM13A and hypoxic responses (21–23). In a cystic fibrosis study, the FAM13A long isoform was detected in undifferentiated primary human basal airway epithelial cells, and *FAM13A* siRNA knockdown modulated RhoA activity, actin stress fiber formation, and EMT (24). However, how these findings relate to COPD pathogenesis remains unknown.

Here, we investigate the expression and physiological function of FAM13A isoforms in organotypic primary human airway epithelial cell cultures and in *Xenopus laevis* embryos, an experimental model for the function of multiciliated cells. In primary human epithelial cells that were differentiated at the air–liquid interface (ALI), we validated a method to separate epithelial cell subsets using surface marker expression and demonstrate that the FAM13A long isoform is predominately expressed in ciliated cells. Fam13a-deficient *X. laevis* embryos demonstrate motility defects that were due to alterations in cilia function, indicating a role for Fam13a in multiciliated cell function. In keeping with this, FAM13A long-isoform deficiency in human airway epithelial cells affected cilia coordination in mucociliary transport assays, without impacting epithelial cell differentiation or ciliogenesis. These data indicate an isoform-specific function for the FAM13A long isoform in the coordination of cilia in mucociliary clearance, which may affect the susceptibility to respiratory diseases such as COPD.

## Methods

For detailed methods, *see* the data supplement.

### Primary Human Bronchial Epithelial Cell (hBEC) Culture

hBECs were expanded in BEGM Bronchial Epithelial Growth Medium (Lonza) with ROCK inhibitor and differentiated at the ALI with Pneumacult-ALI (StemCell Technologies). Gene editing was performed as described previously (25).

### Bulk RNA Sequencing

RNA was sequenced in bulk by Expression Analysis Inc. (Quintiles) and paired-end reads aligned to the human reference genome GRCH38.ensembl86 with STAR. *FAM13A* isoform expression was calculated with Salmon, Version 0.11.0 (26), from an index of Ensembl transcripts (GRCh38 release 94) (19).

### Flow Cytometry and Fluorescence-Activated Cell Sorting

hBEC cultures were dissociated with Accutase (Gibco) before staining. Cells were analyzed with LSRFortessa systems (BD Biosciences) and sorted using MoFlo XDP systems (Beckman Coulter); for imaging flow cytometry, the ImageStream X MKII (Merck) was used.

### Immunocytochemistry

hBEC cultures were fixed in 4% paraformaldehyde or methanol at −20°C and blocked in 1% BSA for 30 min before primary and secondary antibody incubations. *X. laevis* embryos were fixed in MEMFA (MOPS/EGTA/Magnesium Sulfate/Formaldehyde buffer) and blocked in 10% normal goat serum, 0.1% Triton X-100 in PBS before primary and secondary incubations.

### Immunohistochemistry

hBEC cultures were fixed in 10% formalin, embedded in 2% agarose, and then embedded in paraffin and sectioned orthogonally. Sections were stained with hematoxylin and eosin and imaged using an Olympus VS120 slide scanner.

### *X. laevis* Embryo Manipulations and Imaging

*X. laevis* embryos were maintained and staged as described previously (27–29). Morpholinos (MOs) (GeneTools LLC) were injected at the four-cell stage. All animal studies were ethically reviewed and performed in accordance with the Animals (Scientific Procedures) Act 1986 and the GSK Policy on the Care, Welfare and Treatment of Animals. *X. laevis* embryos at Stages 28–32 were imaged for brightfield videos (for analysis details, *see* the data supplement). For bead assays 10-μm FluoSpheres (Invitrogen) were added to the anterior region of the embryos, imaged with a Leica M165FC scope, and analyzed using the Fiji Trackmate plugin (30).

### hBEC Mucociliary Transport Assays

Differentiated hBEC cultures were incubated with 10 mM DTT before the assay. One-micron FluoSpheres Polystyrene Microspheres (ThermoFisher) resuspended in 8% mucin (Sigma) or 2.5% methylcellulose (Merck) were added to the culture surface. Movies of bead movement were taken for 30 seconds at 10 frames per second. (For analysis details, *see* the data supplement.)

### Protein Expression and Purification

FAM13A RhoGAP domain cDNA was cloned into pQE80LNAviH. Biotinylated His_6_-Avi-FAM13A(1–242) was produced in BL21(DH3) *E. coli* and purified using a HisTrap HP column (Cytiva) and S75 16/600 (Cytiva) size exclusion column. RhoA(1–186) F25N in pGEX2T was a gift from Darerca Owen and Helen Mott (University of Cambridge, United Kingdom). GST-RhoA was purified using glutathione sepharose resin (Cytiva). RhoA was cleaved from the GST tag and purified with an S75 16/600 (Cytiva) size exclusion column.

### *In Vitro* GAP Assay

Free P_i_ released by GTP hydrolysis was detected using the EnzChek Phosphate Assay Kit (Invitrogen).

### Active Rho Pulldowns

Plasmids encoding C-terminally 3× FLAG-tagged FAM13A long and short isoforms in pcDNA3.1 were purchased from GenScript Biotech and transiently transfected into HEK293T cells. Pulldowns were performed using an Active Rho Pull-Down and Detection Kit (Thermo Scientific).

## Results

### FAM13A Isoforms Are Differentially Conserved Across Species

Our initial investigations into FAM13A biology led us to compare the *FAM13A* gene across species. Alignment of *FAM13A* homologs in mouse, rat, and frog species demonstrates that mouse and rat express only a short isoform, whereas frog expresses a homolog of the long isoform (Figure 1A). This indicates that the two human isoforms are not conserved across all species and highlights that murine models for dissection of FAM13A function are limited to studying the short isoform only.

### The FAM13A Long Isoform Has RhoGAP Activity toward RhoA

To determine whether the FAM13A long isoform contains a functional RhoGAP domain, the N-terminal domain, containing the putative RhoGAP domain, was purified. This fragment showed GAP activity toward RhoA in an *in vitro* assay (Figure 1B), and overexpression of the full-length FAM13A long isoform reduced active RhoA levels in HEK 293T cells (Figure 1C), indicating that the FAM13A long isoform can act directly as a RhoGAP and reduce RhoA activity.

### FAM13A Isoforms Are Differentially Expressed in Human Lung Cells

*In silico* analysis of *FAM13A* promoters identified by the Functional Annotation of the Mammalian Genome (FANTOM) project indicates that the short *FAM13A* isoform is expressed at a higher level in samples of whole lung or trachea (Figure 1D). However, in bronchial and tracheal epithelial cell samples, expression of the long isoform predominated, indicating that expression of the *FAM13A* long isoform may be relatively higher in lung epithelial cells. The differential expression of the two isoforms may also indicate divergent functions in different cell types.

To investigate the function of FAM13A isoforms, we used ALI cultures of primary hBECs as the current gold-standard, organotypic model of the airway epithelium (31). In this system, basal cells differentiate into multiciliated and secretory cells on air exposure, upregulating expression of markers for secretory cells, multiciliogenesis, and multiciliated cells (*see* Figure E1 in the data supplement). We developed isoform-specific qRT-PCR primers to investigate *FAM13A* expression. In differentiating hBEC cultures, expression of both isoforms increased as differentiation progressed (Figure 1E), indicating a potential role for both FAM13A isoforms in the mature human lung epithelium.

### The FAM13A Long Isoform Is Primarily Expressed in Multiciliated Cells of the Lung Epithelium

**Figure 2.**
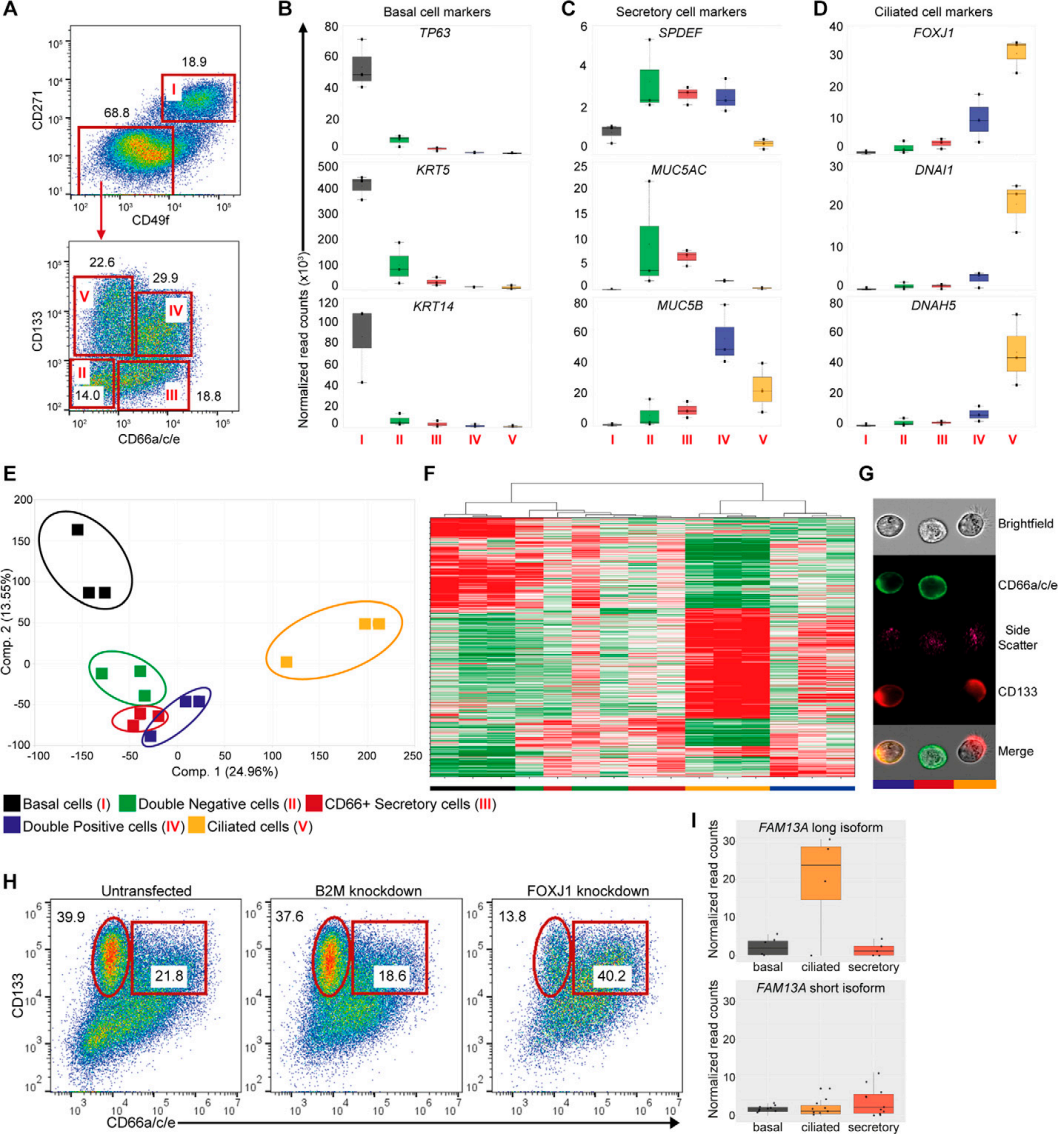
Identification and characterization of epithelial cell subsets from cultures differentiated at the air–liquid interface (ALI). (*A*) Representative flow cytometry dot plots illustrating cell surface expression of CD271, CD49f, CD133, and CD66a/c/e on hBEC ALI cultures at Day 28. The five populations, indicated with red roman numerals, were isolated to homogeneity by fluorescence-activated cell sorting. (*B–D*) Lineage-specific gene expression analysis of sorted epithelial cell subsets by RNA sequencing. Box plots illustrate mean normalized read counts and SD. (*B*) Basal cell markers. (*C*) Secretory cell markers. (*D*) Ciliated cell markers. Distinct subpopulations are color coded in (*B*)–(*G*) and (*I*). (*E*) Principal-component analysis illustrating two components that best define transcriptional territories occupied by epithelial cell subpopulations. (*F*) Heatmap illustrating unsupervised hierarchical clustering of significantly expressed and regulated genes (FC ± 1.5, *P* < 0.05) in epithelial cell subsets, demonstrating lineage relationships. The Double Positive population closely aligns with the ciliated cell populations, suggesting a precursor–product relationship between these populations. (*G*) Representative ImageStream X MKII images of bronchial epithelial cells. CD133 is expressed on ciliated cells and double-positive progenitors. It is interesting that polarization of CD133 is already observed on double-positive progenitor cells. A population of CD66a/c/e single-positive cells is clearly visible. (*H*) Representative dot plots of CD133 and CD66a/c/e expression on unmanipulated, B2M, and FOXJ1 knockdown hBECs. Initial gating was performed as described in (*A*). (*I*) *FAM13A* isoform expression analysis of sorted epithelial cell subsets by RNA sequencing. Box plots illustrate mean normalized read counts and SD. Three donors were used for cell sorting and RNA sequencing in three separate experiments. Two donors in two separate experiments were used in gene-editing studies. FC = fold change.

To better understand the composition of hBECs after ALI differentiation and to map *FAM13A* expression to specific cell populations, we established a flow cytometry–based protocol to distinguish basal, secretory, and multiciliated cells. We used markers that have previously been identified as expressed and regulated at the transcriptional level during epithelial cell differentiation, as well as previously validated cell surface markers (32). Expression of CD271 (NGFR) and CD49f (ITGA6) was used to identify basal cells (Population I), and nonbasal cells were resolved into four distinct cell populations by expression of CD133 (PROM1) and CD66a/c/e (CEACAM1/5/6) (Figure 2A). After sorting, lineage fidelity was established by expression of transcription factors (*TP63*, *SPDEF*, and *FOXJ1*, which encode elements associated with basal, secretory, and multiciliated cells, respectively) and key factors associated with crucial functions of mature epithelial cells (Figures 2B–2D). Population I expressed the highest levels of *TP63*, *KRT5*, *KRT14*, *NGFR*, and *ITGA6* and, therefore, was confirmed as the basal cell population (Figures 2B and E2A). Within the nonbasal cells, the Double Negative population (Population II) expressed genes associated with several epithelial and nonepithelial cell lineages, which suggests that this population is a multipotent progenitor or is heterogenous *de facto* (Figures 2B–2D and E2A). CD133^−^CD66a/c/e^+^ secretory cells (Population III) expressed elements of the secretory cell lineage. The Double Positive population (Population IV) expressed genes associated with secretory cells, club cells, and multiciliogenesis, which suggests a multipotent developmental potential of these cells, previously described in club cells, further underpinned by their transcriptional signatures (Figures 2B–2D and E2A) (33, 34).

The CD133^+^CD66a/c/e^−^ population (Population V) had the highest expression levels of the multiciliated cell–associated transcription factor *FOXJ1* and elements linked to functional cilia, *DNAI1*, and *DNAH5* (Figure 2D). This population occupied a distinct “transcriptional territory” and was completely unrelated to basal or secretory cells (Figures 2E–2F). Cilia were exclusively restricted to this population (Figure 2G), and deletion of *FOXJ1* significantly affected this population (Figure 2H), identifying this as the multiciliated cell population and CD133 as a robust marker of ciliated cells.

Mapping isoform-specific mRNA reads within these populations revealed differential expression of the two *FAM13A* isoforms across the epithelial cell subsets, with the long isoform predominately expressed in multiciliated cells (Figure 2I). This was confirmed by qRT-PCR (Figure E2B). Our data were in keeping with previous single-cell RNA-sequencing analysis that found strong expression of bulk *FAM13A* in ciliated cell populations; however, expression of the two isoforms was not investigated (35). We demonstrate differential *FAM13A* isoform expression in airway epithelial subsets and high *FAM13A* long-isoform expression specifically in multiciliated cells, indicating that FAM13A long-isoform function may be particularly important in this cell type.

### FAM13A Long-Isoform Knockdown in hBECs Does Not Affect Lung Cell Differentiation

**Figure 3.**
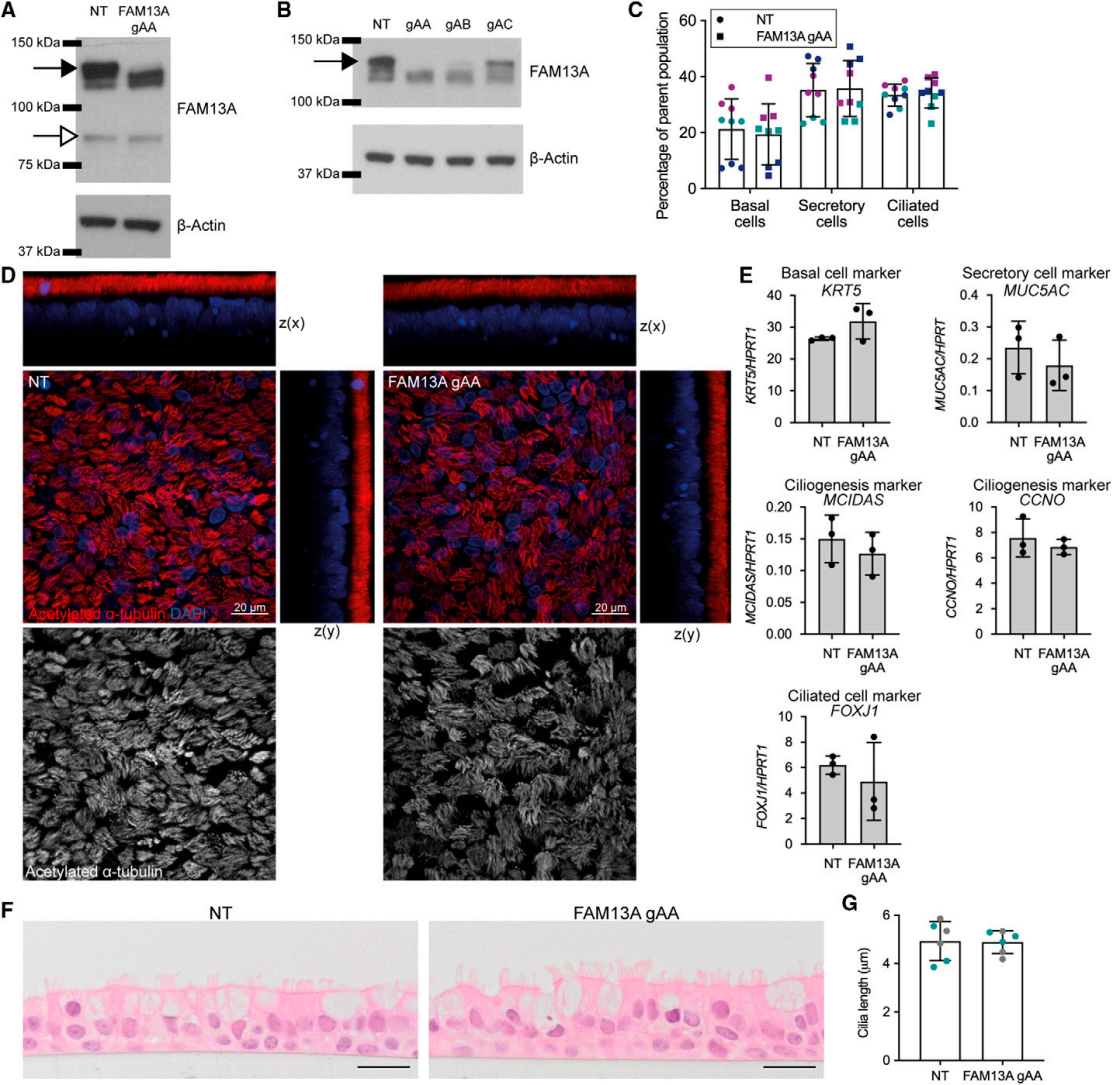
FAM13A long-isoform knockdown does not affect hBEC differentiation. (*A*) FAM13A isoform expression in hBEC cultures edited with nontargeting control (NT) or “FAM13A gAA” CRISPR guide RNA. Long isoform is indicated with a closed arrow, and short isoform is indicated with an open arrow (expected molecular weights were 117 kD and 80 kD, respectively). (*B*) Expression of FAM13A long isoform (closed arrow) protein in hBEC cultures edited with NT or three CRISPR guide RNAs targeting the FAM13A long isoform. (*C*) Flow cytometry analysis of epithelial cell populations in NT and FAM13A gAA cultures. Three independent experiments between Day 25 and Day 31 of ALI culture (annotated in different colors), triplicate cultures per experiment. Data indicate mean ± SD. (*D*) Immunofluorescent staining of cilia (acetylated α-tubulin, red) in NT and FAM13A gAA differentiated hBEC cultures, Scale bars, 20 μm. (*E*) mRNA expression of epithelial cell marker genes in NT and FAM13A gAA differentiated hBECs. Values are normalized to *HPRT1*. Bars represent triplicate cultures from a single donor; results are representative of experiments from two donors, mean ± SD. (*F*) Hematoxylin and eosin staining of NT and FAM13A gAA differentiated hBEC cultures. Scale bars, 20 μm. (*G*) Quantification of cilia length from hematoxylin and eosin staining of NT and FAM13A gAA differentiated hBEC cultures. Two independent cultures of hBECs from different donors, three Transwells per genotype, 100 cilia lengths measured per Transwell. Data indicate mean ± SD; two-way ANOVA, not significant.

Previous analyses of FAM13A function in lung epithelial cells predominately used basal epithelial cells or basal-like cell lines that are unlikely to express the long isoform (11, 13, 14, 16, 17, 22, 24). To dissect the biological functions of the FAM13A long isoform in multiciliated cells, we established a loss-of-function model in differentiated human lung epithelial cells. In primary hBECs, we used CRISPR-Cas9 editing to specifically knockdown the FAM13A long isoform in basal cells followed by differentiation at ALI. FAM13A long isoform expression was almost completely abolished in differentiated hBEC cultures, and the expression of the short isoform was unaffected (Figures 3A, 3B, and E3). Long isoform deficiency did not affect hBEC differentiation: There was no difference in the proportions of basal, secretory, and multiciliated cells between nontargeting control (NT) and FAM13A gAA cells as determined by flow cytometry (Figure 3C), immunofluorescent staining of cilia (Figure 3D), or expression of epithelial cell subset markers (Figure 3E). FAM13A long isoform deficiency also did not affect cilia length in multiciliated cells compared with control cultures (Figures 3F and 3G). This suggests that there may be a role for the FAM13A long isoform in multiciliated cell function rather than differentiation.

### Fam13a Long-Isoform Knockdown in *X. laevis* Embryos Affects Epidermal Cilia Function

**Figure 4.**
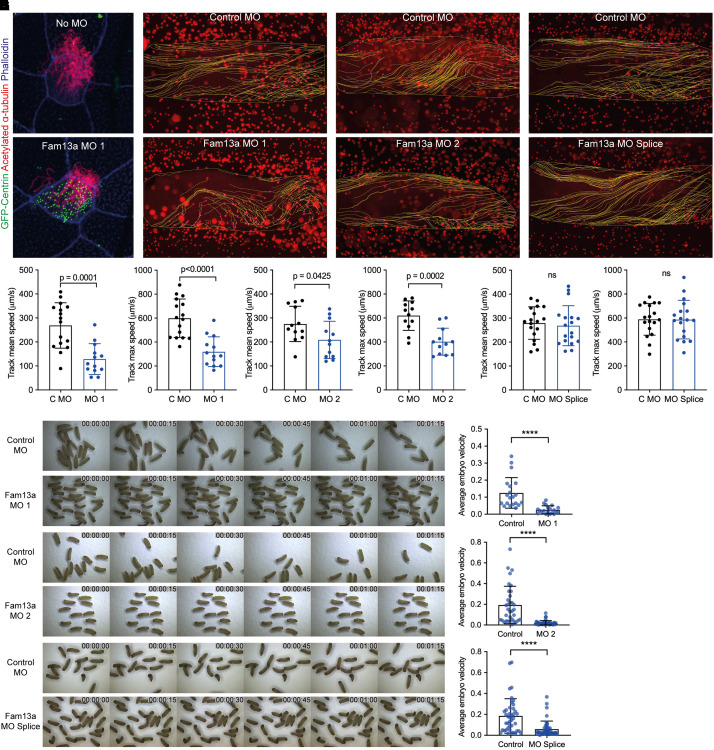
Fam13a long-isoform knockdown in *Xenopus laevis* embryos affects epidermal cilia function. (*A*) Immunofluorescent images of *X. laevis* epidermis after coinjection of morpholino (MO) 1 and GFP-centrin (injection control). (*B–D*) Representative stills from Trackmate analysis of bead movement movies with control and (*B*) MO 1, (*C*) MO 2, or (*D*) MO Splice. (*E–G*) Quantification of Trackmate analysis with control (abbreviated as C) and (*E*) MO 1, (*F*) MO 2, or (*G*) MO Splice. Data indicate mean ± SD, unpaired *t* test. Tracks averaged across each embryo. (*E*) Control, *n* = 1,815 from 16 embryos; MO 1, *n* = 1,990 from 13 embryos; three independent experiments. (*F*) Control, *n* = 1,510 from 12 embryos; MO 2, *n* = 2,018 from 12 embryos; two independent experiments. (*G*) Control, *n* = 2,893 from 18 embryos; MO Splice, *n* = 2,604 from 18 embryos; three independent experiments. (*H–J*) Stills from brightfield movies of *X. laevis* embryo drift and quantification of embryo movement with control and (*H*) MO 1, (*I*) MO 2, or (*J*) MO Splice. Data indicate mean ± SD, unpaired *t* tests with Welch’s correction, *P* < 0.0001. (*H*) Twenty embryos, two independent experiments. (*I*) Control, *n* = 34 embryos; and MO 2, *n* = 43 embryos, three independent experiments. (*J*) Control, *n* = 34 embryos; MO Splice, *n* = 43 embryos; three independent experiments. ****P < 0.001. ns = not significant.

To investigate the function of the FAM13A long isoform in multiciliated cells *in vivo*, we used the *X. laevis* model. This species conserves the RhoGAP-containing isoform of FAM13A and represents a well-established experimental model for ciliogenesis and cilia function (36). To disrupt Fam13a expression, we used MOs that inhibited protein translation (MO 1 and MO 2) or caused alterations in splicing (MO Splice) (*see* Figure E4A). *Xenopus* species have multiciliated cells across their embryonic epidermal surfaces, which developed in all Fam13a MO-treated embryos (Figure 4A). However, tracking of fluorescent beads placed on the embryo surface demonstrated that bead movement was slower across the epithelial surface in embryos treated with translation-blocking MOs (Figures 4B–4G and E4B–E4D), indicating a defect in multiciliated cell function. MO Splice treatment did not reduce fluorescent bead movement, potentially because of an incomplete reduction in the amount of Fam13a protein present in the treated embryos.

Coordinated beating of the epidermal multiciliated cells is known to generate a slow directional movement or drift of early-stage *X. laevis* embryos. Control embryos display the drift phenotype, whereas Fam13a MO-treated embryos do not display this movement (Figures 4H–4J, E4E–E4G, and Videos E1–E6). These results indicate that expression of Fam13a long isoform is required for multiciliated cell function in *X. laevis* epithelia, and there was a more pronounced phenotype with the higher “challenge” of whole-embryo movement compared with fluorescent bead movement.

### FAM13A Long-Isoform Knockdown in hBECs Reduces Cilia Coordination in Mucociliary Clearance Assays

**Figure 5.**
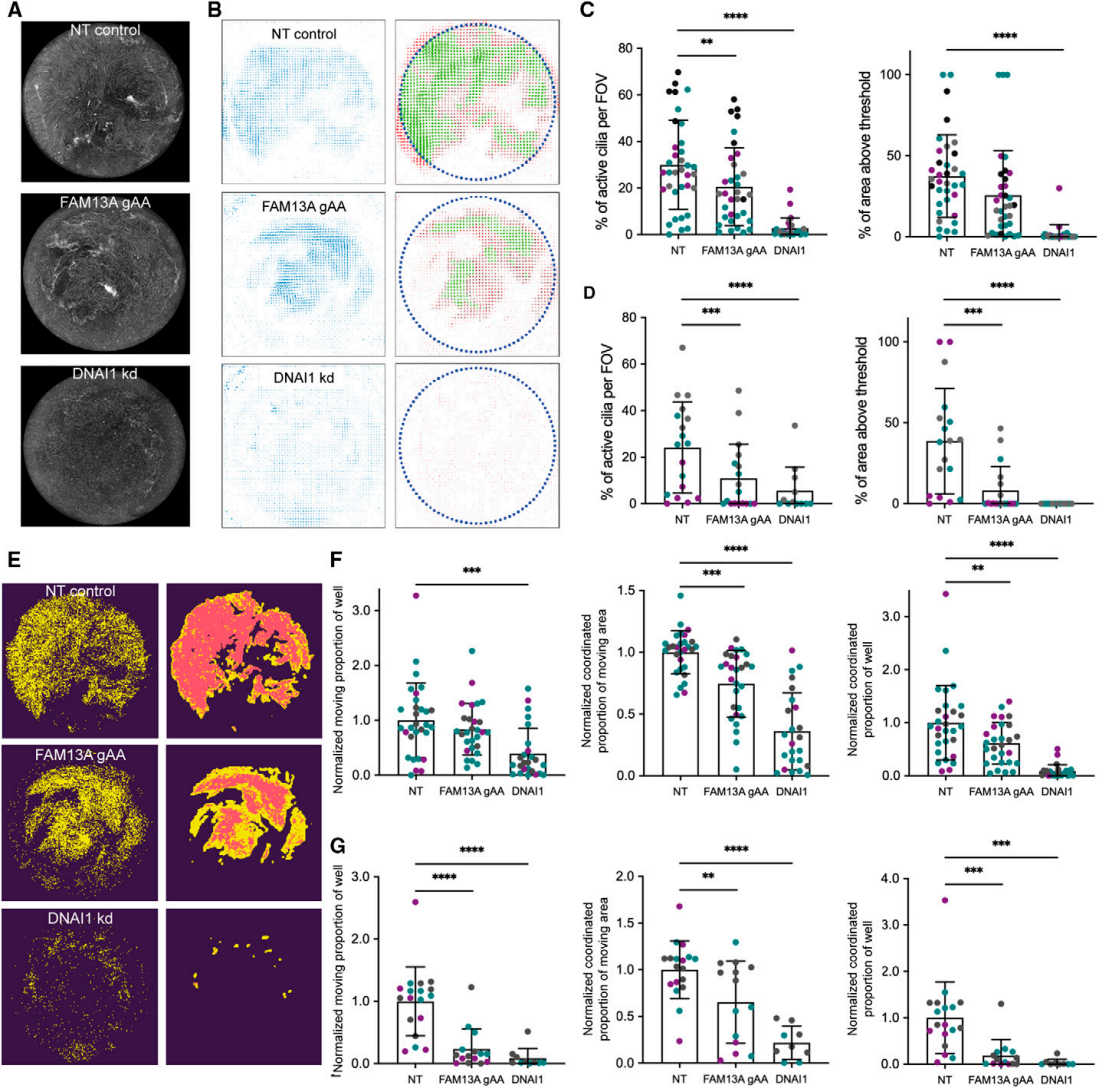
FAM13A long-isoform knockdown reduces cilia coordination in differentiated hBEC mucociliary clearance assays. (*A*) Maximum projections of fluorescent movies of bead movement in 8% mucin on differentiated NT control, FAM13A gAA, or DNAI1 knockdown (kd) hBEC cultures. (*B*) Steps from quantification of hBEC bead assay with the “Cambridge” pipeline. Left: raw flow velocity vector field. Right: final flow velocity vector field: red indicates raw measured velocity vectors, blue dotted line indicates circular mask, black indicates moving cilia, and green indicates vectors with magnitude above threshold. (*C*) Comparison of the percentage of active cilia and percentage of culture above threshold movement in mucociliary transport assays with fluorescent beads in 8% mucin on differentiated NT, FAM13A gAA, or DNAI1 knockdown hBEC cultures using the Cambridge pipeline. Six independent assays, four hBEC donors (indicated with purple, teal, gray, and black), four to six cultures per genotype. Data indicate mean ± SD, two-way ANOVAs. Left graph: **P = 0.0011 and ****P < 0.0001. (*D*) Comparison of the percentage of active cilia and percentage of culture above threshold movement in 2.5% methylcellulose on differentiated NT, FAM13A gAA, or DNAI1 knockdown hBEC cultures using the Cambridge pipeline. Three independent experiments, three hBEC donors (indicated with purple, teal, and gray), four to six cultures per genotype. Data indicate mean ± SD, two-way ANOVAs. Left graph: ***P = 0.0006. Right graph: ***P = 0.0003 and ****P < 0.0001. (*E*) Steps from quantification of hBEC bead assay with the “Stevenage” pipeline. Left: pixels that change in intensity over the course of the experiment were used to form the moving mask. Right: moving and coordinated masks overlaid. (*F*) Comparison of moving and coordinated regions of bead movement in mucociliary transport assays with fluorescent beads in 8% mucin on differentiated NT, FAM13A gAA, or DNAI1 knockdown hBEC cultures using the Stevenage pipeline. Five independent experiments, three hBEC donors (indicated with purple, teal, and gray), four to six cultures per genotype. Data indicate mean ± SD, two-way ANOVAs. Left graph: ***P = 0.0003. Middle graph: ***P = 0.0002 and ****P < 0.0001. Right graph: **P = 0.0068 and ****P < 0.0001. (*G*) Comparison of moving and coordinated regions of bead movement in mucociliary transport assays with fluorescent beads in 2.5% methylcellulose on differentiated NT, FAM13A gAA, or DNAI1 knockdown hBEC cultures using the Stevenage pipeline. Three independent assays, three hBEC donors (indicated with purple, teal, and gray), four to six cultures per genotype. Data indicate mean ± SD, two-way ANOVAs. Left graph: ****P < 0.0001. Middle graph: **P = 0.0040 and ****P < 0.0001. Right graph: ***P = 0.0006 (bottom) and 0.0005 (top). Two-way ANOVA factors were defined as experiment and genotype. The *P* values quoted here correspond to the *P* values associated with the effect of variation between genotypes. FOV = field of view.

To probe multiciliated cell function in hBECs, we analyzed mucociliary transport efficiency by following the movement of fluorescent beads across the surface of NT and FAM13A long-isoform knockdown cultures (Figure 5A and Videos E7–E9). DNAI1, a respiratory cilia dynein complex component, is important for cilia movement, and DNAI1 knockdown served as a positive control for the disruption of cilia function (*see* Figure E5A). Fluorescent beads were applied to the apical surface of differentiated hBEC cultures in one of two viscous media (8% mucin or 2.5% methylcellulose) and imaged for 30 s. These concentrations were selected to challenge the cilia function of the cultures as a more striking phenotype was seen in our *X. laevis* model with the greater challenge of whole-embryo movement.

To quantify the movement of beads across the surface of the cultures, we used two independently optimized analysis pipelines referred to as the “Cambridge” and “Stevenage” pipelines. Both pipelines were applied to assays from at least three hBEC donors and demonstrated a defect in bead movement in FAM13A long-isoform knockdown cultures (Figures 5B–5G). The Cambridge pipeline, using a particle image velocimetry approach to measure the speed and directionality of the bead movement, identified a significant decrease in the percentage of active cilia upon FAM13A long-isoform knockdown in both media (Figures 5B–5D), and in the amount of movement above the threshold velocity in 2.5% methylcellulose (Figure 5D). The Stevenage pipeline complemented this analysis, using a different approach to define moving regions of the Transwell before identifying directionality of bead movement and analyzing alignment (or coordination) of nearby regions. FAM13A long-isoform knockdown cultures demonstrated a significant reduction in the amount of moving area per culture in 2.5% methylcellulose (Figure 5G), and a significant reduction in the area of the cultures where bead movement was coordinated in both viscous media (Figures 5E–5G). Together, these analyses indicate that although there are no changes in the number of multiciliated cells in differentiated FAM13A knockdown hBEC cultures, the cilia in these cultures have defects in their ability to generate flow in viscous conditions.

## Discussion

COPD-associated *FAM13A* SNPs are a major genetic risk factor for development of the disease. Their presence in introns suggests a regulatory nature, indicating that aberrant FAM13A expression contributes to COPD pathogenesis. Two distinct isoforms are produced at the human *FAM13A* locus, suggesting potentially divergent functions in normal physiology. A tendency for increased overall *FAM13A* mRNA expression has been demonstrated for several COPD-associated risk alleles (3, 5–7). However, it is important to note that these studies have been conducted on whole-lung tissue, and the two *FAM13A* isoforms—which may be differentially impacted by genetic variants—have not been distinguished. In human lung epithelial cell ALI cultures, we described a flow cytometry surface marker panel to separate distinct epithelial cell subsets and demonstrated that the FAM13A long isoform is primarily expressed in multiciliated cells, whereas the short isoform is expressed to a lower level throughout the epithelial cell subsets. It is interesting to note that other COPD-associated genes, such as *ARMC2*, have similarly cell-specific expression patterns (37, 38). Our data demonstrating that individual isoforms are expressed differently in lung epithelial cell types highlight that we cannot conclude the true impact of COPD-associated SNPs on *FAM13A* expression unless individual isoforms are measured in isolated cell subsets from genotyped donors. Therefore, in light of these new findings, the common interpretation that *FAM13A* expression is increased by COPD-associated SNPs needs to be reconsidered. In addition, the expression pattern of FAM13A isoforms indicates the potential for distinct mechanisms across cell types, which may combine to affect COPD pathogenesis.

Previous publications have focused on the function of Fam13a in mouse models, which are genetic models for the short isoform only, and few studies have focused on the role of the long isoform specifically. Here, we demonstrate that the FAM13A long isoform is mainly expressed in the multiciliated cell subset of the human lung epithelium. Therefore, we established approaches to investigate the function of FAM13A in the human airway epithelium and in the *Xenopus* model in which the long isoform is conserved.

In *Xenopus* embryos, an *in vivo* model for multiciliogenesis, Fam13a knockdown did not affect the development of multiciliated cells at the epidermal cell surface but did affect cilia movement, as demonstrated by the reduced flow of fluorescent beads across the embryo surface and reduced whole-embryo drifting phenotype. The drift motion is generated by the coordinated polarized movement of the epidermal ciliated cells, suggesting a loss in coordination of cilia beating on multiciliated cells in Fam13a knockdown *Xenopus* epidermis.

In human lung epithelial cell ALI cultures, specifically disrupting expression of FAM13A long isoform using CRISPR-Cas9 gene editing did not impact the differentiation of multiciliated cells or other epithelial cell subsets, and cilia length was also unaffected. This indicated that expression of FAM13A long isoform in multiciliated cells is not required for multiciliogenesis.

In our hBEC mucociliary transport assays, we used 8% mucin or 2.5% methylcellulose to provide two independent types of high-viscosity environment in which to test ciliary beating under stringent conditions. Against this challenge, we found a reduction in cilia function in FAM13A long isoform–specific knockdowns in both media. We quantified this by using two independent image analysis pipelines. The “Cambridge” pipeline demonstrated a significant reduction in the number of active cilia per field of view and a reduction in the amount of the culture where bead speed is greater than the mean control speed. The “Stevenage” pipeline demonstrated a significant difference between the fraction of the culture moving between control and FAM13A long-isoform knockdown in 2.5% methylcellulose and a significant reduction in the amount of the cultures where this movement is coordinated in both media. This suggests that the directionality or efficacy of cilia movement is impacted in long isoform–specific knockdowns, which, in turn, affects the speed of bead movement. Together with the *Xenopus* data, this suggests that expression of FAM13A long isoform is important to ensure the coordination of cilia movement in epithelial cells, and SNPs that alter FAM13A isoform expression may affect the efficiency of mucociliary clearance in the human lung.

We used purified protein to show, for the first time, that the FAM13A putative RhoGAP domain directly activates GTPase activity of RhoA. These biochemical experiments directly demonstrate RhoGAP activity of FAM13A long isoform and extend prior, indirect evidence showing, by overexpression, that the FAM13A long isoform can act as a RhoA modulator and affect arrangement of the actin cytoskeleton (24). Underlying the basal bodies of the cilia is a highly interconnected array of microfilaments, including actin filaments and microtubules (39, 40). RhoA activity is important for actin arrangement and has been implicated in early ciliogenesis in mouse tracheal cells (41). Deficiency of the FAM13A long isoform did not affect ciliogenesis in hBEC ALI cultures, but expression of FAM13A long isoform increased during hBEC cell differentiation, specifically in multiciliated cells. We hypothesize that FAM13A RhoGAP activity controls ciliary coordination by regulating the structure of the underlying actin cytoskeleton.

To our knowledge, this is the first demonstration that the two FAM13A isoforms are differentially expressed between airway epithelial cell subsets. The FAM13A long isoform is predominantly expressed in multiciliated cells of the lung epithelium, suggesting a role for FAM13A RhoGAP activity in multiciliated cells. FAM13A long-isoform deficiency in *Xenopus* embryos and human primary airway epithelial cells demonstrated a crucial role for the long isoform in cilia coordination, which is required for the polarization of mucociliary clearance. Airway multiciliated cells represent a central component of the respiratory tract frontline defense, and multiciliated cell dysfunction has been reported in the context of COPD (42). Therefore, our work describing a physiological function for the FAM13A long isoform in the regulation of cilia function has clear relevance to COPD pathogenesis, as any dysregulation of this function could underlie the increased susceptibility to lung diseases such as COPD in the presence of *FAM13A* risk alleles, especially when compounded with the effects of environmental risk factors. Further work focusing on expression patterns and functions of the two FAM13A isoforms in COPD versus healthy individuals will be important to fully understand the physiological role of FAM13A isoforms as well as the link between *FAM13A* SNPs and the risk of developing COPD.
